# Prognostic impact of genetic alterations and methylation classes in meningioma

**DOI:** 10.1111/bpa.12970

**Published:** 2022-02-25

**Authors:** Anna S. Berghoff, Thomas Hielscher, Gerda Ricken, Julia Furtner, Daniel Schrimpf, Georg Widhalm, Ursula Rajky, Christine Marosi, Johannes A. Hainfellner, Andreas von Deimling, Felix Sahm, Matthias Preusser

**Affiliations:** ^1^ Division of Oncology Department of Medicine I Medical University of Vienna Vienna Austria; ^2^ Division of Biostatistics German Cancer Research Center (DKFZ Heidelberg Germany; ^3^ Institute of Neurology Medical University of Vienna Vienna Austria; ^4^ Department of Biomedical Imaging and Image‐guided Therapy Medical University of Vienna Vienna Austria; ^5^ Clinical Cooperation Unit Neuropathology German Cancer Consortium German Cancer Research Center Heidelberg Germany; ^6^ Department of Neuropathology Institute of Pathology University Hospital Heidelberg Heidelberg Germany; ^7^ Department of Neurosurgery Medical University of Vienna Vienna Austria

**Keywords:** meningioma, methylation classes, mutation, prognosis

## Abstract

Meningiomas are classified based on histological features, but genetic and epigenetic features are emerging as relevant biomarkers for outcome prediction and may supplement histomorphological evaluation. We investigated meningioma‐relevant mutations and their correlation with DNA methylation clusters and patient survival times. Formalin‐fixed and paraffin‐embedded samples of 126 meningioma patients (WHO grade I 52/126; 41.3%; WHO grade II: 48/126; 38.1%; WHO grade III: 26/126; 20.6%) were investigated. We analyzed *NF2, TRAF7, KLF4, ARID, SMO, AKT,* *TERT* promotor, *PIK3CA*, and *SUFU* mutations using panel sequencing and correlated them to DNA methylation classes (MC) determined using 850k EPIC arrays. The TRAKL mutation genotype was characterized by the presence of any of the following mutations: *TRAF7, AKT1*, and *KLF4*. Survival data including progression‐free survival (PFS) and overall survival (OS) was retrieved from chart review. Mutations were evident in 90/126 (71.4%) specimens with mutations in *NF2* (39/126; 31.0%), *TRAF7* (39/126; 31.0%) and *KLF4* (25/126; 19.8%) being the most frequent ones. Two or more mutations were observed in 35/126 (27.8%) specimens. While *TRAKL* was predominantly found in benign MC, NF2 was associated with malign MC (*p* < 0.05). *TRAF7, KLF4*, and TRAKL mutation genotype were associated with improved PFS and OS (*p* < 0.05). *TERT* promotor methylation, intermediate, and malign MC were associated with impaired PFS and OS (*p* < 0.05). Methylation cluster showed better prognostic discrimination for PFS and OS (c‐index 0.77/0.75) than each of the individual mutations (c‐index 0.63/0.68). In multivariate analysis correcting for age, gender, MC, and WHO grade, none of the individual mutations except TERT remained an independent significant prognostic factor for PFS. Molecular profiling including mutational analysis and DNA methylation classification may facilitate more precise prognostic assessment and identification of potential targets for personalized therapy in meningioma patients.

## Graphical abstract

Molecular profiling including meningioma relevant mutations and DNA methylation classification may facilitate more precise prognostic assessment and identification of potential targets for personalized therapy in meningioma patients.

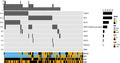

**Key points**

- Meningiomas are heterogeneous in terms of prognosis, even within a given WHO grade, requiring a prognosis adapted therapeutic approach.
- Molecular markers have been suggested to improve the accuracy of outcome prediction, but the number of studies on DNA methylation is still limited and the reports on the prognostic role of mutations are conflicting.
- We validate the association of meningioma relevant mutations as well as methylation classes with clinical parameters.
- Methylation classes, *TRAF7, KLF4, NF2, TERT* promoter mutations, and TRAKL mutation type were associated with progression‐free survival.
- In order to assess the power of the so far proposed markers, we enriched our cohort for, first, higher‐grade meningioma (WHO grade II and III) as the impact of adjuvant radiation is particularly controversially discussed among these, and second, on WHO grade I subtypes prone to harbor the TRAKLS mutation genotype.

## 1 INTRODUCTION

Meningiomas are the most common primary intracranial tumors. Although the majority of cases have a benign clinical course, aggressive cases with impaired overall survival exist and require adaption of the therapeutic approach (1). Diagnostic difficulties are common in meningioma as diversity of histological characteristics and biological behavior is a key feature. Different histological patterns can co‐occur within the same sample, challenging the diagnostic interpretation and the resulting prognostic assessment as the basis for therapeutic approaches (2, 3). Overall the current edition of the WHO classification defines 15 different meningioma subtypes: 9 variants of WHO grade I meningiomas, which are on average associated with slow growth rate and benign biological behavior; 3 histological variants of WHO grade II meningiomas characterized by an increased risk of recurrence; 3 histological variants of WHO grade III meningiomas, which are associated with an aggressive clinical course and high recurrence rates (4). While the prognostic role of WHO grading for outcome prediction is evident on a cohort‐basis, single patients can have clinical courses divergent from grading. Importantly, the WHO grade is currently the basis for post‐neurosurgical treatment decisions: additional radiotherapy can be considered in higher grade meningiomas in order to prevent local recurrence (1). However, adjuvant radiation is associated with side effects and should only be applied if a clinically relevant progression risk exists. Recently, several separate studies identified genetic alterations associated with the clinical course of meningiomas as a basis for more precise diagnostic assessment (5). Single mutations of *AKT1, TRAF7, KLF4*, and *SMO* as well as the TRAKLS mutation genotype (defined by the presence of one of the following: *SMO, AKT1, KLF4,* *TRAF7* mutation, or a combination of AKT1/TRAF7 of KLF4/TRAF7) was shown to be associated with clinical factors and occur typically in WHO grade I meningiomas (6, 7). While *AKT1* and *SMO* mutations were shown to be associated with rather impaired progression‐free survival in some studies (8, 9) a study investigating the full TRAKLS mutation genotype showed them to be associated with favorable progression‐free survival (10). Meningiomas with mutant *NF2* are more likely to be atypical than meningioma of the TRAKLS group (7, 8, 11). Further, the incidence of *TERT* promotor mutations was shown to be higher in recurrent and higher grade meningiomas as well as associated with shorter progression‐free survival (12, 13). Recently, these genetic aberrations were correlated with methylation classes (MC) and a methylation‐based tumor classification as the basis for future diagnosis and treatment of meningioma has been proposed (14, 15, 16). Here, we investigated the correlation of meningioma‐relevant mutations with MC and the clinical course in a retrospective series of meningiomas.

## 2 METHODS

### 2.1 Patient cohort

Patients with histologically proven meningioma diagnosis were identified from the Neuro‐Biobank, Institute of Neurology, Medical University of Vienna. We enriched our cohort for, first, higher‐grade meningioma (WHO grade II and III) as the impact of adjuvant radiation is particularly controversially discussed among these, and second, for WHO grade I subtypes prone to harbor the TRAKLS mutation genotype (6, 7, 17). All specimens were investigated by a board‐certified Neuropathologist to confirm histological diagnosis. Formalin‐fixed paraffin‐embedded (FFPE) material was screened macroscopically for sufficient quantity and microscopically for tumor cell content. Clinical data including histological diagnosis, WHO grading, progression, and survival times were retrieved by chart review. Progression/recurrence was defined based on the written report of the radiology consultant and documented in the patient file. Re‐evaluation of magnet resonance images (MRI) was not possible as most patients received the cranial MRI outside the center. Cranial re‐staging was performed 3 months after surgery followed by another MRI 6 months later and followed by one MRI per year unless symptoms occur. If no recurrence or progression is evident after 5 years re‐staging intervals are extended to 2 years. Only patients with complete follow‐up data were included. The study was approved by the local ethics committee of the Medical University of Vienna with the approval number 078/2004.

### 2.2 Methylation classes and panel sequencing

Methylation analysis using 850k EPIC (Illumina, San Diego, CA, USA) results were available from a previous analysis and performed as described (14). Further, panel sequencing for genes reported to impact meningioma namely *NF2, TRAF7, KLF4, SMO, AKT1,* *TERT* promotor, *ARID,* *SUFU, and PIK3CA*, was performed using the previously published methods (14). Libraries were generated based on a hybrid‐capture enrichment panel and sequenced on an Illumina NextSeq 500 in paired end‐mode (12). All exome or near exome (splice‐site) genetic variations were included while intron sequences except the *TERT* promoter, and polymorphisms with >1/100 000 incidence in databases were excluded. Germline DNA was not available. Single‐nucleotide variants and small insertion/deletions left after these filtering criteria are subsequently termed “mutation” in the text. The TRAKLS mutation genotype was defined by the presence of at least one of the following mutations: *TRAF7, AKT1,* *KLF4*, or/and *SMO* (10). *TERT* promotor mutations C228T and C250T were combined in one group. Further, *ARID1A, ARID1B, and ARID2* mutation were combined in the *ARID* mutation group. See Table S1 for detailed information of the exact mutations. Source data of the present manuscript is not publicly available.

### 2.3 Statistical analysis

Methylation classes were defined using unsupervised clustering. Importantly, the classes were available from a previous publication and not newly defined (14). Fisher's exact test was used to assess group differences in categorial variables. Progression‐free survival (PFS) was defined as months from meningioma surgery to radiological diagnosis of progression/ recurrence or death, whichever occurred first. Patients were censored at last info on progression. Overall survival (OS) was defined as time to death. Patients were censored at last info on survival status. Distribution of survival times was estimated by Kaplan‐Meier method, and log‐rank test was used to compare groups. Cox proportional hazards model was applied for univariable and multivariable analysis of PFS and OS. For each mutation, a separate multivariable Cox model was fitted adjusting for WHO grade, age, sex, and methylation cluster. Firth correction was used in case of complete separation. Harrell's concordance index (c‐index) was used to assess predictive discrimination. *p* values of 0.05 or less were considered significant. Due to the exploratory and hypothesis generating design of the present study no adjustment for multiple testing was applied (18).

## 3 RESULTS

### 3.1 Patients characteristics

One hundred twenty‐six meningioma specimens of 126 patients [94/126 (74.6%) female] with a median age of 59 years (range 6–86 years) at meningioma surgery were available for analysis. Median PFS was 27 months with 32 events. For OS the median follow‐up time was 101 months with 27 deaths and a 5‐year survival rate of 83%. Of 39 patients with WHO grade 2 meningioma, 27/39 (69.2%) presented with atypical meningioma and 12/39 (30.7%) with other rare types of WHO grade II meningioma. PFS (*p* = 0.890) and OS (*p* = 0.150) did not differ between atypical meningioma and other rare types of WHO grade II meningioma. Table 1 list further patients’ characteristics.

**TABLE 1:** Patients’ characteristics

Characteristic	Entire cohort (n = 126)	
	n	%
Age at diagnosis, years (range)	59.0 (6–86)	
Gender		
Male	32	25.4
Female	94	74.6
Histology		
Anaplastic meningioma	25	19.8
Atypical meningioma	36	28.6
Chordoid meningioma	12	9.5
Secretory meningioma	24	19.0
Rhabdoid meningioma	1	0.8
Psammomatous meningioma	21	16.7
Microcystic meningioma	3	2.4
Transitional meningioma	4	3.2
WHO grading		
I	52	41.3
II	48	38.1
III	26	20.6
Localization		
Convexity	10	7.9
Basal	28	22.2
Frontal	21	16.7
Occipital	3	2.4
Posterior fossa	6	4.8
Parietal	3	2.4
Temporal	3	2.4
Spinal	10	7.9
Missing	42	33.3
Progression/deaths (PFS events)		
Yes	32	25.3
No	94	74.6
Median progression‐free survival, months (range)	27 (13–36)	
Alive at last follow up		
Yes	99	78.6
No	27	21.4
Median survival from meningioma surgery, months (range)	101 (90–112)	

### 3.2 Presence of meningioma relevant mutations

**FIGURE 1:**
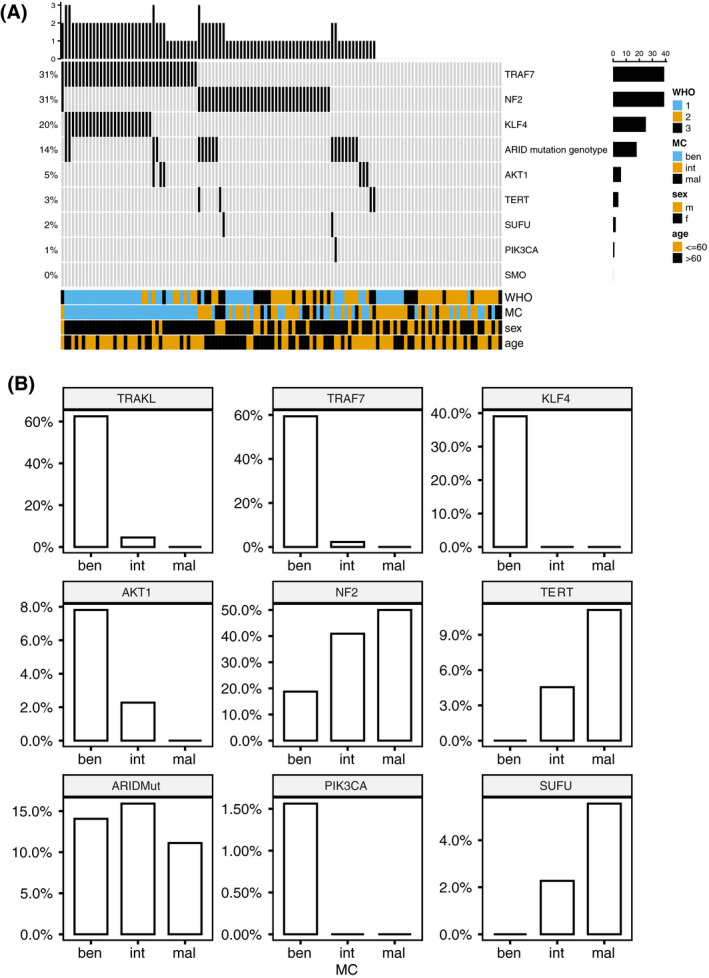
Frequency of meningioma relevant mutations (A) and methylation classes (B)

Ninety of 126 (71.4%) meningioma specimens presented with at least one meningioma relevant mutation, while no mutations could be detected in 36/126 (28.6%) meningioma specimens. The most frequently affected genes were *NF2* (39/126; 30.9%) and *TRAF7* (39/126; 30.9%) followed by *KLF4* (25/126; 19.8%) and one of the *ARID* genes (18/126; 14.3%). *AKT1* (6/126; 4.8%), *TERT* promoter (4/126; 3.2%), *SUFU* (2/126; 1.6%), and *PIK3CA* (1/126; 0.8%) mutations however were only infrequently observed. *SMO* mutations were absent in the analyzed cohort (Figure 1A).

Two or more mutations were evident in 40/126 (31.7%) meningioma specimens. Due to the absence of *SMO* mutations, we included only patients with the presence of either one of the following mutations in the TRAKL mutation genotype: *TRAF7, AKT1,* *KLF4* (10). The TRAKL mutation genotype was evident in 42/126 (33.3%) specimens. The co‐occurrence of *TRAF4* and *KLF4* mutations was the most frequently observed combination as all patients with *KLF4* mutations also presented with *TRAF4* mutation (*p* < 0.001). *NF2* mutations were almost mutually exclusive with the TRAKL mutation genotype as only one patient presented with an overlap (*p* < 0.001).

### 3.3 Correlation of meningioma relevant mutations with methylation classes

The presence of meningioma relevant mutations was further correlated with methylation classes as previously described (14). The frequency of methylation classes in the present cohort is displayed in Table 2 and Figure 1B.

**TABLE 2:** Methylation classes and presence of meningioma relevant mutations

	Entire cohort (n = 126)	
	n	%
Methylation classes		
MC benign	64	51
MC intermediate	44	35
MC malignant	18	14
Meningioma relevant mutations		
NF2	39	30.9
TRAF7	39	30.9
KLF4	25	19.8
ARID	18	14.3
AKT1	6	4.8
TERT promotor	4	3.2
PIK3CA	1	0.8
SUFU	2	1.6
SMO	0	0

TRAKL mutation genotype significantly more frequently observed in the benign MC (62.5%) than in the intermediate (4.5%) or the malignant MC (0%; *p* < 0.001). The *KLF4* and the *TRAF7* mutations was also more common among the benign MC (39.1%; 59.4%) than in the intermediate (0%; 2.3%) or the malignant MC (0%; 0%; *p* < 0.001). Consequently the TRAKL mutation genotype was more common among the benign MC (62.5%) than in the intermediate (4.5%) or the malignant MC (0%; *p* < 0.001). *NF2* mutations were significantly more frequently observed in the malign MC (50.0%) than in the benign (18.8%) and the intermediate MC (40.9%; *p* < 0.001). Further, *TERT* promotor mutations were more frequently observed in malign MC (11.1%) than in the benign (0%) and the intermediate MC (4.5%; *p* < 0.04). No significant association with MC and *AKT1*, ARID mutation genotype, *PIK3CA*, or *SUFU* mutation was observed (*p* > 0.05).

### 3.4 Correlation of meningioma relevant mutations and methylation class with progression‐free and overall survival

**FIGURE 2:**
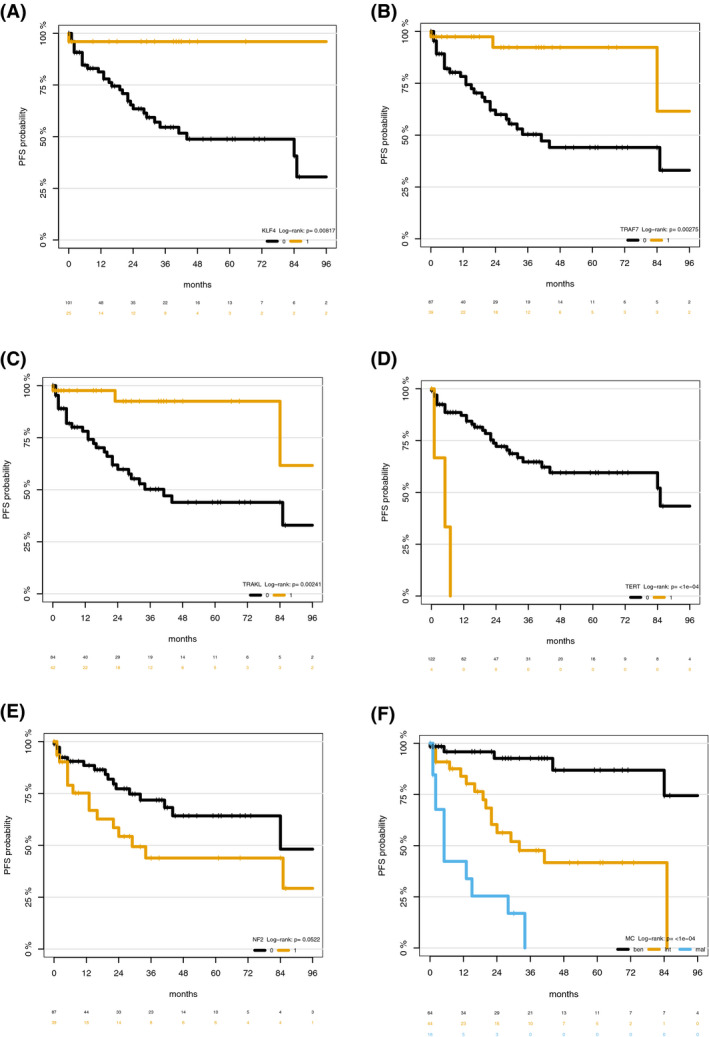
Progression‐free survival according to the presence of *KLF4* mutation (A), *TRAF7* mutation (B), *NF2* mutation (C), *TERT* promotor mutation (D), *TRAKLS* mutation genotype (E), and methylation class (F)

**FIGURE 3:**
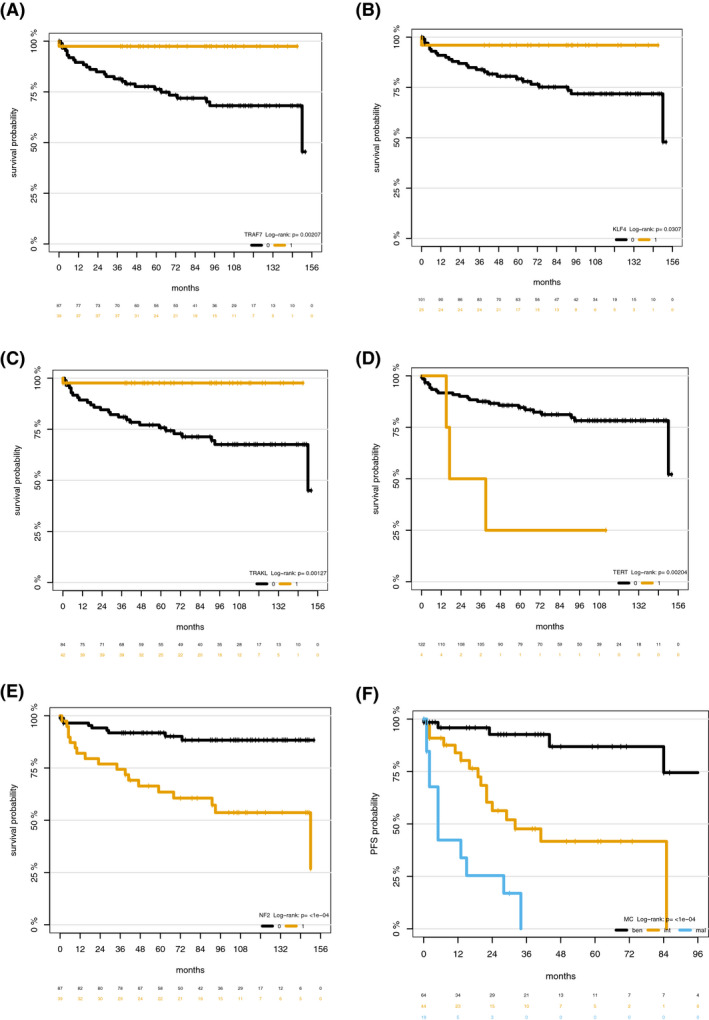
Overall free survival according to the presence of *KLF4* mutation (A), *TRAF7* mutation (B), *NF2* mutation (C), *TERT* promotor mutation (D), *TRAKLS* mutation genotype (E), and methylation class (F)

All meningioma‐relevant mutations with sufficient prevalence were tested for association with progression‐free survival and overall survival. In univariable analysis presence of *TRAF7* and *KLF4* mutation as well as the TRAKL mutation genotype were associated with improved PFS and OS prognosis with 5‐year PFS and OS rates of at least 90% and 95%, respectively (*p* < 0.05; Tables 3 and 4; Figures 2A–C and 3A–C). *NF2* and *TERT* promotor mutation were associated with impaired PFS and OS prognosis with a median PFS of 29 and 5 months, and 5‐year OS rates of 63% and 25% (*p* < 0.05; Tables 3 and 4; Figures 2D,E and 3D,E). Methylation classes, WHO grading, and age at diagnosis were associated with PFS and OS (Figures 2F and 3F; *p* < 0.05).

**TABLE 3:** Univariate Cox regression analysis and c‐index for progression‐free survival

	Hazard ratio	95% CI	*p* value	c‐index	
					*p*‐value for comparison with MC
Methylation class				0.77	
Benign	Reference				
Intermediate	6.25	1.92–10.76	<0.001		
Malignant	22.94	7.45–70.63	<0.001		
KLF4	0.11	0.01–0.81	0.03	0.57	<0.001
TRAF7	0.20	0.06–0.64	0.001	0.62	<0.001
NF2	1.98	0.98–3.99	0.06	0.59	<0.001
TERT promotor	12.13	3.32–44.30	<0.001	0.55	<0.001
TRAKL genotype	0.19	0.06–0.63	0.01	0.63	<0.001
ARID mutation	0.97	0.37–2.85	0.95	0.52	<0.001
AKT1	1.60	0.01–12.35	0.76	0.51	<0.001
Panel (TRAKL + NF2 + TERT)				0.68	0.052
Age (per 10 year increase)	1.36	1.00–1.85	0.049	0.63	0.03
WHO grade				0.69	0.055
I	Reference				
II	1.07	0.40–2.85	0.90		
II	4.71	2.01–11.06	<0.001		

**TABLE 4:** Univariate Cox regression analysis and c‐index for overall survival

	Hazard ratio	95% CI	*p* value	c‐index	
					*p*‐value for comparison with MC
Methylation class				0.75	
Benign	Reference				
Intermediate	4.80	1.56–14.79	0.01		
Malignant	13.26	4.15–42.42	<0.001		
KLF4	0.15	0.02–1.10	0.06	0.58	<0.001
TRAF7	0.08	0.01–0.63	0.016	0.64	0.003
NF2	4.67	2.09–10.44	<0.001	0.68	0.23
TERT promotor	5.45	1.62–18.33	0.01	0.55	<0.001
TRAKL genotype	0.08	0.01–0.63	0.01	0.65	0.01
ARID mutation	1.03	0.36–3.00	0.95	0.50	<0.001
AKT1	0.44	0.00–3.12	0.51	0.52	<0.001
					
Panel (TRANKL +NF2+TERT)				0.74	0.86
Age (per 10 year increase)	1.94	1.36–2.76	<0.001	0.71	0.53
WHO grade				0.76	0.90
I	Reference				
II	3.03	0.82–11.24	0.10		
II	15.04	4.32–54.39	<0.001		

Methylation cluster showed better prognostic discrimination for PFS and OS (c‐index 0.77/0.75) then each of the individual mutations (c‐index 0.63/0.68; Tables 3 and 4). Further, methylation cluster showed better prognostic discrimination for PFS than a model based on the sequencing panel (*TRAKL, NF2,* *TERT*; c‐index 0.69; *p* = 0.052) but not for OS (c‐index 0.74; *p* > 0.05; Tables 3 and 4). In comparison to WHO grading, methylation cluster showed a better prognostic discrimination for PFS (0.77 vs. 0.69; *p* = 0.055) but not for OS (0.75 vs. 0.76; *p* > 0.05; Tables 3 and 4). In multivariable analysis, only *TERT* promotor mutation (HR 4.34; 95% CI 1.08–17.42; *p* = 0.04) but none of the other individual mutations remained an independent prognostic factor for PFS when adjusting for age, sex, MC, and WHO grade. Further, none of the individual mutations remained an independent prognostic factor for OS when adjusting for age, sex, MC, and WHO grade (*p* > 0.05). In contrast, MC always remained a significant prognostic factor for both PFS and OS (*p* < 0.05).

## 4 DISCUSSION

Meningiomas can be clinically challenging in modern neuro‐oncology, as the selection of patients is essential for personalized and risk‐adapted treatment planning. Here, we validate that distinct prognostic subgroups can be defined by the presence of molecular driver mutations and methylation classes (14). Future clinical treatment trials should consider the inclusion of molecular information in order to investigate the therapeutic potential in distinct meningioma subgroups.

Meningioma‐relevant mutations were present in 90/126 (71.4%) specimens including *NF2, TRAF7, KLF4, SMO, AKT1,* *TERT* promotor, *ARID,* *SUFU, and PIK3CA* mutations in similar frequencies compared to previous studies (6, 11, 12, 14, 19, 20, 21). In line with previous publications, we could validate the overlap of certain meningioma relevant mutations such as *AKT1* and *KLF4* with *TRAF7* mutations (19, 21). The TRAKLS mutation genotype as well as *TERT* promotor, *KLF4*, and *TRAF7* mutations presented in our cohort with statically significant association with survival prognosis, as shown in previous independent cohorts (10, 11, 12). A recent study of 469 meningiomas suggested a 22x higher recurrence rate in aggressive subgroups (NF2, PI3K, HH, TRAF7) compared to others (KLF4, POLR2A, SMARCB1) (22). Further, KLF4K mutations were shown to cause HIF pathways up‐regulation as a potential new therapeutic avenues (23). The present cohort also provided the previously described strong association of *KLF4*/*TRAF7* mutations and secretory subtype, while the association of *AKT1* or *SMO* mutations with skull base localization and meningothelial histology was not significant in our series, possibly due to the limited number of affected cases. Importantly, an entire mutation panel is necessary to determine genetic distinct subgroups of meningioma, as certain overlaps exist but are rarely mutually exclusive in a cohort containing WHO grade I to III meningiomas (6, 10, 11, 12). Furthermore, we could validate that methylation classes correlate significantly with the presence of specific meningioma relevant mutations, as well as with clinical characteristics including progression‐free survival (14). Indeed, analysis of methylation classes provides a promising method for diagnostic brain tumor work‐up in addition to routine histological analysis as it might reveal certain prognosis relevant molecular alterations (24). As expected, WHO grade was also associated with survival time in our cohort, thus underscoring the importance of histological features for prognostic evaluation. However, co‐occurrence of several histological features within the same specimen may introduce bias and inaccuracy (4, 25). Indeed, WHO grading was recently shown to suffer from suboptimal inter‐observer reproducibility and little prognostic effect in higher grade meningiomas (26). Genetic and epigenetic analysis could help to give an more objective, reliable, and reproducible prognostic assessment (5, 14).

We selected for higher‐grade meningioma (WHO grade II and III) as well as less common histology subtypes as the impact of adjuvant radiation is particularly controversially discussed in this cohort with high recurrences rates up to 39%–58% (1, 4). The ROAM/EORTC‐1308 trial currently investigates whether early adjuvant radiotherapy reduces the risk of tumor recurrence following complete surgical resection of atypical meningioma (17). The WHO classification of meningioma currently faces discussions due to the wide range of observed clinical behavior of WHO grade I and II meningiomas (1). Therefore, expansion of the prognostic work up seems of particular interest in order to provide a molecular marker driven stratification in future clinical trials. Indeed, molecular characteristics including meningioma‐relevant mutations and methylation classes could be used in future trials to re‐define patient populations of particular risk for local relapse and enable a risk‐adapted therapeutic approach in meningioma in order to avoid both, over‐ and undertreatment in a personalized context (5).

Although we were able to validate the importance of meningioma‐relevant mutations and their association with methylation classes and survival times our data set has to face some limitations. A considerable limitation is certainly that we were not able to predefine progression/recurrence uniformly. Data on progression was retrieved by retrospective chart review and central re‐assessment of the neuro‐imaging was not possible. Due to the frequent performance of MRI images outside the center only the written statement was available, the original MRI was not available, and, therefore, the recently established response assessment guidelines could not be applied (27). However, our survival data profits from the high patient adherence at our centers as none of the patients were lost to follow up.

Nevertheless, we aimed to contribute to the clarification of the role of TRAKLS mutations and to compare these with previous findings on the correlation of defined methylation classes and meningioma‐relevant mutations. Here, we could validate the role of TRAKLS mutations as being correlated with outcome in our large, independent dataset, but also detected the superior prognostic role of MCs. Thereby, the data support the basis for the concept ‘integrated’ diagnosis as proposed in the revision of the WHO 2016 classifications for CNS tumors also for meningioma (4). This further adds to previous studies suggesting DNA methylation pattern as predictor of outcome in meningiomas (15, 28, 29). Based on the previously published discovery set, we here were able to stratify for six biological (MC ben‐1, 2, 3, int‐A, B, mal) and three combined clinical MCs (benign, intermediate, malignant) (14). Further, in contrast to the previously conducted studies, we could correlate genetic alterations to the particular methylation profiles gaining a more comprehensive insight on the molecular alterations driving meningioma recurrence. Nevertheless, further studies are needed to investigate the value of meningioma relevant mutation or methylation classes as a stratification factor in prospective clinical trials.

In conclusion, we were able to validate the prognostic impact as well as the correlation with clinical characteristics of the most frequent meningioma‐relevant mutations, and correlated these markers with methylation classes, which could be used in future clinical trials for patient stratification.

## CONFLICT OF INTEREST

Anna Sophie Berghoff has research support from Daiichi Sankyo and honoraria for lectures, consultation, or advisory board participation from Roche Bristol‐Meyers Squibb, Merck, Daiichi Sankyo as well as travel support from Roche, Amgen and AbbVie. Matthias Preusser has received honoraria for lectures, consultation, or advisory board participation from the following for‐profit companies: Bayer, Bristol‐Myers Squibb, Novartis, Gerson Lehrman Group (GLG), CMC Contrast, GlaxoSmithKline, Mundipharma, Roche, BMJ Journals, MedMedia, Astra Zeneca, AbbVie, Lilly, Medahead, Daiichi Sankyo, Sanofi, Merck Sharp & Dome, Tocagen. The following for‐profit companies have supported clinical trials and contracted research conducted by Matthias Preusseer with payments made to his institution: Böhringer‐Ingelheim, Bristol‐Myers Squibb, Roche, Daiichi Sankyo, Merck Sharp & Dome, Novocure, GlaxoSmithKline, AbbVie. All other authors report no conflict of interest concerning this specific publication. FS: Speakers’ bureau Illumina, Agilent, Medac, SAB AbbVie.

## AUTHOR CONTRIBUTIONS

Anna S. Berghoff: study design, data collection, data interpretation, manuscript writing, approval of final manuscript version. Thomas Hielscher: data collection, data interpretation, manuscript writing, approval of final manuscript version. Gerda Ricken: data collection, data interpretation, manuscript writing, approval of final manuscript version. Julia Furtner: data collection, data interpretation, manuscript writing, approval of final manuscript version. Daniel Schrimpf: data collection, data interpretation, manuscript writing, approval of final manuscript version. Georg Widhalm: data collection, data interpretation, manuscript writing, approval of final manuscript version. Ursula Rajky: data collection, data interpretation, manuscript writing, approval of final manuscript version. Christine Marosi: data collection, data interpretation, manuscript writing, approval of final manuscript version. Johannes A. Hainfellner: data collection, data interpretation, manuscript writing, approval of final manuscript version. Andreas von Deimling: data collection, data interpretation, manuscript writing, approval of final manuscript version. Felix Sahm: study design, data collection, data interpretation, manuscript writing, approval of final manuscript version. Matthias Preusser: study design, data collection, data interpretation, manuscript writing, approval of final manuscript version.

## Supporting information


**Table S1** Detailed information of the exact mutations in the analyzed meningioma cohort

## Data Availability

Data is available from the corresponding author upon request.
